# Personality Traits and Emotional Support Exchanges Among Oldest-Old Parents and Older-Adult Children in Korea

**DOI:** 10.1093/geroni/igaa057.1261

**Published:** 2020-12-16

**Authors:** Kyuho Lee

**Affiliations:** Daegu University, Gyeongsan, Kyongsang-bukto, Republic of Korea

## Abstract

Previous studies show that personality traits are predictors of individuals’ exchanges (i.e., giving, perceiving, and evoking) in social support in general relationships. Less attention has been paid, however, to the roles of personality traits in parent-child relation, especially in the very old parent-child dyads. Focusing on personality’s effects on the perception of received support, this study examines 1) whether personality traits are associated with a perception of received emotional support, 2) which personality traits work as a predictor of support perception, and 3) whether the similarity between parent and children exist in the patterns of personality-support relationships among Korean very old parent-child dyads. A total of 105 dyads of very old parents, 81 to 97 years old (M = 87.9, SD = 2.8), and their older-adult children, 65 to 72 years old (M = 65.9, SD = 1.2), participated in the study. The results of the actor-partner interdependence model reveal that less neurotic and more agreeable parents perceived more emotional support from children(actor-effects); parents with more agreeable children perceived more support from children(partner-effect). There was no actor- and partner effects of personality traits on the emotional support children perceived. Our findings show that children, as compared to their parents, are maybe less affected by parents’ personality traits reflected by parents’ interaction behaviors. We further provide explanations of each path from the personality traits to emotional support.

